# Global, regional, and national burden of type 1 diabetes in adolescents and young adults

**DOI:** 10.1038/s41390-024-03107-5

**Published:** 2024-03-05

**Authors:** Boshen Gong, Wanyu Yang, Yumin Xing, Yaxin Lai, Zhongyan Shan

**Affiliations:** Department of Endocrinology and Metabolism, Institute of Endocrinology, NHC key Laboratory of Diagnosis and Treatment of Thyroid Diseases, The First Affiliated Hospital of China Medical University, Shenyang, Liaoning 110001 P. R. China

## Abstract

**Background:**

Type 1 diabetes (T1D) incidence in adolescents varies widely, but has increased globally in recent years. This study reports T1D burden among adolescents and young adults aged 10–24-year-old age group at global, regional, and national levels.

**Methods:**

Based on the Global Burden of Disease Study 2019, we described the burden of T1D in the 10–24-year-old age group. We further analyzed these trends by age, sex, and the Social Development Index. Joinpoint regression analysis was used to assess temporal trends.

**Results:**

T1D incidence among adolescents and young adults increased from 7·78 per 100,000 population (95% UI, 5·27–10·60) in 1990 to 11·07 per 100,000 population (95% UI, 7·42–15·34) in 2019. T1D mortality increased from 5701·19 (95% UI, 4642·70–6444·08) in 1990 to 6,123·04 (95% UI, 5321·82–6887·08) in 2019, representing a 7·40% increase in mortality. The European region had the highest T1D incidence in 2019. Middle-SDI countries exhibited the largest increase in T1D incidence between 1990 and 2019.

**Conclusion:**

T1D is a growing health concern globally, and T1D burden more heavily affects countries with low SDI. Specific measures and effective collaboration among countries with different SDIs are required to improve diabetes care in adolescents.

**Impact:**

- We assessed trends in T1D incidence and burden among youth in the 10–24-year-old age group by evaluating data from the Global Burden of Disease Study 2019.
- Our results demonstrated that global T1D incidence in this age group increased over the past 30 years, with the European region having the highest T1D incidence.
- Specific measures and effective collaboration among countries with different SDIs are required to improve diabetes care in adolescents.

## Introduction

The increasing burden of type 1 diabetes (T1D) in adolescents and young adults is a major healthcare concern worldwide.^1^ T1D incidence in childhood and adolescence is steadily rising and now stands at 22·9 new cases per year per 100,000 children up to the age of 15 years in Germany.^2^ T1D burden has been attributed to rapid economic development and urbanization. The cost of diabetes care is at least 3·2 times greater than the average per capita healthcare expenditure, rising to 9·4 times in the presence of complications.^3^ A multicenter study in the US showed that the overall unadjusted estimated incidence rates of T1D in youths increased by 1·4% annually from 2002 to 2012.^4^ Thus, understanding the global burden of T1D in adolescents is important for the optimal utilization of healthcare resources in different countries.

Children are more sensitive to a lack of insulin than adults and are at a higher risk of rapid development of diabetic ketoacidosis.^5^ Prior studies have indicated geographic differences in T1D trends. A multicenter prospective registration study conducted in 26 European centers reported significant increases in T1D incidence among adolescents between 1989 and 2013.^6^ A cross-sectional multicenter study of 3.47 million youths (aged 19 years or younger) in the US found a significant increase in the estimated prevalence of T1D, from 1·48 cases per 1000 youths to 2.15 cases per 1000 youths.^7^ The International Diabetes Federation Atlas 10th edition reported that T1D incidence in children and adolescents varies widely, and is increasing in many nations.^8^ In our study, we investigated the global burden and the most substantial changes in the trend of T1D in adolescents and young adults aged 10–24 years. Many countries, particularly those with a low or middle social development index (SDI), lack high-quality information regarding T1D trends in adolescents. Thus, there is an urgent need to characterize T1D burden in adolescents and provide more information to local governments to ease this burden. The Global Burden of Disease (GBD) Study 2019 is an international collaboration that offers an opportunity to analyze disease trends on a global scale. In this study, we aimed to analyze global trends in T1D prevalence, incidence, disability-adjusted life-year (DALY), and mortality rates among adolescents across every decade since 1990, based on the latest data from GBD 2019.

## Methods

GBD 2019 provides the most up-to-date estimation of the descriptive epidemiological data including incidence, prevalence, mortality, and disability adjusted life years (DALY) on a total of 369 diseases and injuries for 204 countries and territories from 1990 to 2019.^9^ All countries and territories were classified into 21 regions according to epidemiological similarities, and could be grouped into six regions (African region, Eastern Mediterranean region, European region, Region of the Americas, South-East Asia region, and Western Pacific region) by the WHO.^10^ The number of incident cases, prevalent cases, deaths, and DALYs were extracted from GBD 2019. All disease estimated from GBD contains 95% uncertainty intervals (UI) for each metric, which are based on the 25th and 97.5th values of 1000 draws of the posterior distribution. Rates in our study are shown per 100 000 population. Details of data inputs, processing, synthesis, and final model are available in the accompanying GBD 2019 publications.^9^ The GBD database had data on T1D for different age periods. The WHO defined adolescence is the phase of life between childhood and adulthood, from ages 10 to 19. It is a unique stage for adolescents to lay the foundations of good health.^11^ Thus, we defined younger adolescents ages 10-14years as age subgroups, ages 15–19 could be defined older adolescent, and 20 to 24 young adults.^12^ To comprehensively describe the global trend of T1D in the life phase of adolescents and young adults, we defined younger adolescent (aged 10–14 years), older adolescent (aged 15–19 years), and young adults (aged 20–24 years). Data were collected from the GBD 2019 database in three age groups (10–14 years, 15–19 years, and 20–24 years), and both sexes.

The GBD 2019 analyzed the Socio-demographic index (SDI) for each country, which is an indicator estimated as a compositive of income per capita, average years of schooling among adults aged 15 and older, and fertility rate in female under 25 years old. All countries and territories were grouped into five categories based on the SDI (low SDI, low-middle SDI, middle SDI, high-middle SDI, and High SDI).

### Analysis

We conducted comparisons between the sexes, age groups (three intervals; 10–14, 15–19, and 20–24 years), SDI (five categories), and WHO regions (six regions). Incidence, prevalence, death, and DALYs were reported with 95% UI to eliminate the effects caused by differences in population structures. Then, we used the Joinpoint Regression Analysis to identify the substantial changes in trends of above indicators, and we used a maximum of 5 Joinpoints as the option of analysis. We evaluated the incidence, prevalence, DALYs, and mortality trends in various countries and regions based on average annual percent change (AAPC) by Joinpoint regression analysis.^13^ The Joinpoint regression model was used to subsection describe disease trends from 1990 to 2019, and found out if the junctions of different segments had statistically significant. Countries with missing or zero values in their decade data were excluded from the analysis because a Joinpoint regression could not be conducted in this circumstance. The value of the AAPC is computed as a weighted average of the annual percentage change (APC) values in the regression analysis. The approximate 95% CI for AAPC was calculated by the empirical quantile method. We calculated the AAPCs between 1990 and 1999, between 2000 and 2009, between 2010 and 2009, and between 1990 and 2019. All analyses were performed using RStudio software (version R-4.2.2), and Joinpoint Regression Program (version 4.9.1.0).

## Results

### Global burdens of T1D in adolescents and young adults

**Fig. 1:**
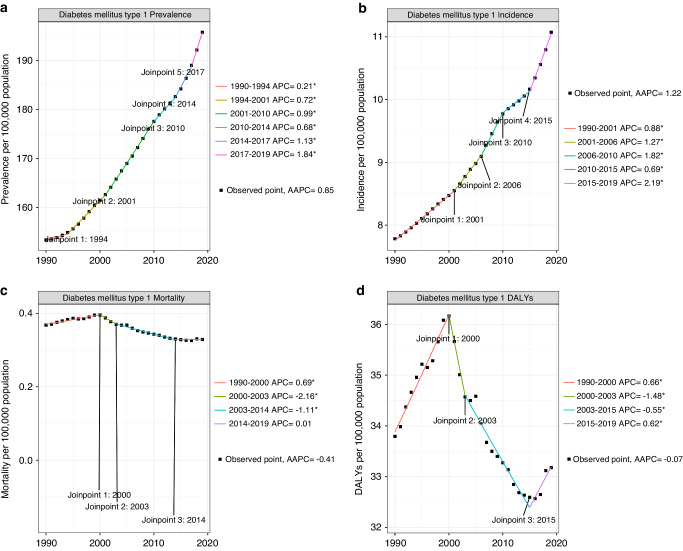
Global trends in T1D among adolescents by Joinpoint regression analysis (1990-2019). Joinpoint regression analysis of global T1D prevalence (**a**), incidence (**b**), mortality (**c**), and DALYs (**d**) in adolescents and young adults aged 10–24 years from 1990 to 2019. DALY disability-adjusted life year, AAPC average annual percentage change, APC annual percentage change.

From 1990 to 2019, the incidence and prevalence of T1D in adolescents and young adults showed an overall increasing trend (Table 1). T1D incidence increased between 1990 and 1999 (AAPC, 0·88 [95% confidence interval (CI), 0·86–0·91]) and rose rapidly between 2000 and 2009 (AAPC, 1·41 [95% CI, 1·32–1·50]). The overall incidence rate in 1990 (7·78) cases per 100,000 population [95% uncertainty interval (UI), 5·27–10·60] increased to 2019 (11·07 cases per 100,000 population [95%] UI, 7·42–15·34; AAPC, 1·22 [95% CI, 1·18–1·27]). The overall global prevalence of T1D increased from 2,376,444 (95%UI 1,761,701–3,073,758) in 1990 to 364,4613 (95%UI, 2,655,059–4,756,336) in 2019, representing a 53·36% increase over the 30-year period (Table 2). Joinpoint regression analysis showed that the increasing trend of T1D incidence could be divided into six periods: 1990–1994, 1994–2001, 2001–2010, 2010–2014, 2014–2017, and 2017–2019 (Fig. 1).

**Table 1:** Global AAPCs in prevalence, incidence, mortality, and DALYs of type 1 diabetes among adolescents and young adults aged 10–24 years.

	Prevalence		Incidence		Mortality		DALYs	
	AAPC (95%CI)	*P* value	AAPC (95%CI)	*P* value	AAPC (95%CI)	*P* value	AAPC (95%CI)	*P* value
1990–1999	0.49 (0.46–0.52)	<0.001	0.88 (0.86–0.91)	<0.001	0.69 (0.56–0.82)	<0.001	0.66 (0.56–0.76)	<0.001
2000–2009	0.96 (0.94–0.97)	<0.001	1.41 (1.32–1.50)	<0.001	–1.46 (–1.99 to –0.93)	<0.001	–0.86 (–1.25 to -0.47)	<0.001
2010–2019	1.09 (1.04–1.14)	<0.001	1.35 (1.27–1.44)	<0.001	–0.48 (–0.69 to –0.29)	<0.001	–0.03 (–0.20 to 0.14)	0.70
1990–2019	0.85 (0.82–0.87)	<0.001	1.22 (1.18–1.27)	<0.001	–0.41 (–0.6 to –0.22)	<0.001	–0.07 (–0.21 to 0.07)	0.32

*AAPC* average annual percentage change.

**Table 2:** The incidence and DALYs of T1D in adolescents and young adults and their AAPCs from 1990 to 2019.

	Incidence Cases(n), 1990	Incidence (per100, 000),1990	Cases(n), 2019	Incidence (per100, 000),2019	AAPC 1990- 2019	*P* value	DALYs Cases(n), 1990	DALYs (per100, 000),1990	Cases(n), 2019	DALYs (per100, 000),2019	AAPC 1990- 2019	*P* value
Global	120580.28 (81681.43–164256.68)	7.78 (5.27– 10.60)	206136.12 (138076.08–) 285590.97	11.07 (7.42– 15.34)	1.22 (1.18–1.27)	<0.001	523450.60 (427184.50– 612813.80)	33.79 (27.58– 39.56)	617763.70 (519442.00– 745712.50)	33.12 (27.87– 39.50)	–0.07 (–0.21 to 0.07)	0.319
Sex												
Boy	65053.33 (44514.79–87999.81)	8.26 (5.66– 11.18)	111120.71 (75271.16– 153830.34)	11.65 (7.89– 16.13)	1.19 (1.16–1.23)	<0.001	251585.80 (215984.4– 294670.60)	31.96 (27.44– 37.43)	330516.10 (283199.40– 393945.40)	34.66 (29.70– 41.32)	0.28 (0.12– 0.43)	<0.001
Girl	55526.95 (37333.90– 75781.23)	7.29 (4.90– 9.95)	95015.41 (63469.04– 132623.84)	10.46 (6.99– 14.60)	1.25 (1.21– 1.30)	<0.001	271864.80 (201148.90–325322.50)	35.69 (26.40– 42.70)	287247.60 (227931.20– 354293.50)	31.62 (25.09– 39.00)	-0.43 (–0.57 to –0.28)	<0.001
Age group												
10–14	57980.13 (28692.33– 87272.01)	10.80 (5.35– 16.26)	88164.44 (41482.76– 138154.81)	13.73 (6.46– 21.51)	0.82 (0.80– 0.84)	<0.001	136517.40 (97813.82– 170497.00)	25.44 (18.23– 31.77)	152957.70 (124300.45– 191466.00)	23.82 (19.36– 29.81)	–0.23 (–0.48 to 0.03)	0.081
15–19	34369.55 (19126.19– 56494.06)	6.61 (3.68– 10.87)	60683.28 (30834.45– 103668.24)	9.79 (4.98– 16.73)	1.36 (1.29– 1.43)	<0.001	168928.90 (129619.20– 202152.80)	32.51 (24.95– 38.91)	190187.00 (155409.30– 232630.50)	30.70 (25.08– 37.55)	–0.22 (–0.39 to –0.04)	0.013
20–24	28230.60 (13301.84– 50920.09)	5.73 (2.70– 10.34)	57288.40 (25267.81– 105339.49)	9.55 (4.21– 17.55)	1.78 (1.65– 1.91)	<0.001	218004.30 (180144.80– 253858.20)	44.25 (36.56– 51.53)	274619.00 (233819.40– 323196.40)	45.76 (38.96– 53.85)	0.14 (0.04–0.24)	0.007
High SDI	27468.29 (20037.19– 35519.86)	14.98 (10.93– 19.37)	37801.25 (25548.33– 51837.42)	21.53 (14.55– 29.53)	1.25 (1.17– 1.33)	<0.001	56803.84 (44495.81– 73159.90)	30.98 (24.27–39.90)	57138.41 (42480.20– 77584.30)	32.55 (24.20– 44.20)	0.17 (0.04–0.31)	0.011
High-middle SDI	21051.01 (14429.57– 28383.53)	6.96 (4.77– 9.38)	28264.31 (18823.22– 39142.10)	11.13 (7.42– 15.43)	1.62 (1.52– 1.71)	<0.001	77262.10 (66825.12– 90045.66)	25.53 (22.08– 29.76)	57740.96 (46829.31– 73637.47)	22.76 (18.45– 29.02)	–0.40 (–0.63 to –0.17)	0.001
Middle SDI	27857.50 (18233.77– 38777.48)	5.12 (3.35– 7.12)	47143.02 (31503.53– 65653.87)	8.58 (5.73– 11.95)	1.79 (1.74– 1.84)	<0.001	174503.10 (143910.00– 199509.60)	32.05 (26.43– 36.65)	156815.00 (136218.40– 184668.20)	28.54 (24.79– 33.60)	–0.39 (–0.48 to –0.30)	<0.001
Low- middle SDI	28235.64 (18558.25–40050.93)	7.98 (5.24– 11.32)	52951.27 (35629.43– 74459.36)	10.37 (6.98– 14.59)	0.91 (0.88– 0.93)	<0.001	144027.20 (106709.30– 174473.00)	40.70 (30.15– 49.30)	197939.80 (161406.00– 236242.30)	38.78 (31.62– 46.28)	–0.18 (–0.42 to 0.06)	0.136
Low SDI	15915.79 (10524.40– 22525.94)	9.71 (6.42- 13.75)	39875.50 (26451.31– 56774.37)	10.73 (7.12– 15.28)	0.35 (0.33– 0.36)	<0.001	70402.24 (53602.41– 86704.98)	42.97 (32.71– 52.92)	147576.65 (122213.59– 176362.02)	39.73 (32.90– 47.47)	–0.27 (–0.38 to –0.15)	<0.001
African Region	16326.67 (10923.64– 22624.87)	10.05 (6.73– 13.93)	37910.93 (25266.10– 53176.98)	10.60 (7.07– 14.87)	0.18 (0.17– 0.2)	<0.001	66423.63 (52374.94– 80144.24)	40.90 (32.25– 49.35)	131036.06 (106849.95– 158209.31)	36.65 (29.88– 44.25)	–0.37 (–0.40 to –0.34)	<0.001
Eastern Mediterranean Region	10968.68 (7330.123– 15410.19)	9.12 (6.09– 12.81)	29878.47 (19980.87– 41523.84)	14.14 (9.46–19.65)	1.53 (1.50– 1.56)	<0.001	41397.65 (34433.86– 49855.80)	34.41 (28.62– 41.44)	91335.14 (73356.26– 111946.07)	43.23 (34.72– 52.99)	0.84 (0.68– 0.99)	<0.001
European Region	21941.74 (16166.53– 28492.39)	11.18 (8.24– 14.52)	30597.84 (20400.42– 42148.90)	18.80 (12.54– 25.90)	1.81 (1.76– 1.86)	<0.001	57449.11 (48827.71– 68997.61)	29.28 (24.88– 35.16)	53310.14 (42114.12– 69033.33)	32.76 (25.88– 42.42)	0.43 (0.24– 0.62)	<0.001
Region of the Americas	24556.96 (16907.70– 32930.71)	12.42 (8.55– 16.66)	32893.13 (22174.40– 45509.49)	14.22 (9.58– 19.67)	0.48 (0.33– 0.62)	<0.001	70019.43 (58651.44– 85012.57)	35.42 (29.67– 43.00)	84560.53 (68572.15– 103422.09)	36.55 (29.64– 44.70)	0.12 (0.01–0.23)	0.031
South-East Asia Region	30786.00 (20313.20– 43537.54)	7.69 (5.07– 10.87)	56046.20 (37675.64– 78946.40)	10.02 (6.74– 14.12)	0.90 (0.79– 1.01)	<0.001	165706.00 (116577.80– 202826.90)	41.38 (29.11– 50.65)	194448.10 (158204.20– 234613.50)	34.77 (28.29– 41.95)	–0.65 (–1.02 to –0.27)	0.001
Western Pacific Region	15735.80 (10406.83– 21553.19)	3.36 (2.22– 4.61)	18317.39 (12041.64– 25550.49)	5.47 (3.59– 7.63)	1.69 (1.58– 1.81)	<0.001	121385.64 (96420.94– 141798.73)	30.78 (25.76– 35.66)	61813.34 (52382.74– 73737.63)	24.61 (20.83– 28.77)	–1.17 (–1.43 to –0.91)	<0.001

Data in parentheses are 95% uncertainty intervals for cases, incidences, and DALYs, and 95% CIs for AAPCs.

*AAPC* average annual percentage change.

From 1990 to 2019, T1D mortality and DALY rates initially exhibited an increasing trend (1990–1999), followed by a subsequent decline (Table 3). Joinpoint regression analysis identified a substantial change in both T1D mortality (in 2000, 2003, and 2014) and DALY (in 2000, 2003, and 2015) rates. T1D incidence and prevalence increased significantly during the 30-year period. The mortality rate in 2019 (0·33 deaths per 100,000 population [95% UI, 0·29–0·37]) was lower than that in 1990 (0·37 deaths per 100,000 population [95% UI, 0·30–0·42]; AAPC, –0·41 [95% CI, –0·60 to –0·22]). The global DALY rate significantly decreased after 2000, with the most notable decline observed between 2000 and 2003.

**Table 3:** The prevalence and morality of T1D in adolescents and young adults and their AAPCs from 1990 to 2019.

	Prevalence cases(n), 1990	Prevalence (per 100, 000),1990	Cases(n), 2019	Prevalence (per 100, 000),2019	AAPC 1990– 2019	*P* value	Mortality Cases(n), 1990	Mortality (per 100, 000),1990	Cases(n), 2019	Mortality (per 100, 000),2019	AAPC 1990– 2019	*P* value
Global	2376444.00 (1761701.00– 3073758.00)	153.42 (113.73– 198.44)	3644613.00 (2655059.00– 4756336.00)	195.75 (142.60– 255.46)	0.85 (0.82– 0.87)	<0.001	5701.19 (4642.7– 6444.08)	0.37 (0.30– 0.42)	6123.04 (5321.82– 6887.08)	0.33 (0.29– 0.37)	–0.41 (–0.60 to –0.22)	<0.001
Sex												
Boy	1206677.00 (891833.40– 1571014.00)	153.30 (113.30– 199.58)	1871433.00 (1360287.70– 2446297.00)	196.27 (142.66– 256.56)	0.85 (0.83– 0.88)	<0.001	2715.58 (2363.19– 3161.72)	0.34 (0.30– 0.40)	3371.90 (2999.90– 3835.35)	0.35 (0.31– 0.40)	0.08 (–0.14 to 0.29)	0.489
Girl	1169767.00 (865724.60– 1507234.00)	153.55 (113.64– 197.85)	1773180.00 (1288764.30– 2325674.00)	195.20 (141.87– 256.02)	0.83 (0.80– 0.86)	<0.001	2985.61 (2088.39– 3516.95)	0.39 (0.27– 0.46)	2751.14 (2144.16– 3262.54)	0.30 (0.24– 0.36)	–0.87 (–1.00 to –0.74)	<0.001
Age group												
10–14	632465.20 (428098.80–868397.00)	117.85 (79.77– 161.81)	919560.00 (596551.80– 1299626.20)	143.19 (92.89– 202.38)	0.67 (0.66– 0.69)	<0.001	1379.38 (926.76– 1742.41)	0.26 (0.17– 0.32)	1409.11 (1140.52– 1705.13)	0.22 (0.18– 0.27)	–0.53 (–0.69 to –0.36)	<0.001
15–19	822623.60 (602732.10– 1082017.00)	158.32 (116.00– 208.24)	1243928.60 (884515.50– 1653485.00)	200.78 (142.77– 266.89)	0.82 (0.79– 0.85)	<0.001	1777.20 (1332.84– 2056.67)	0.34 (0.26– 0.40)	1770.94 (1477.14– 2023.97)	0.29 (0.24– 0.33)	–0.64 (–0.88 to –0.40)	<0.001
20–24	921355.00 (706223.20–1165346.00)	187.01 (143.34– 236.53)	1481124.70 (1116022.80– 1915825.00)	246.79 (185.96– 319.23)	0.96 (0.91– 1.01)	<0.001	2544.61 (2071.77– 2873.06)	0.52 (0.42– 0.58)	2942.99 (2577.75– 3298.66)	0.49 (0.43– 0.55)	–0.14 (–0.29 to 0.00)	0.05
High SDI	645961.50 (511844.70– 787027.50)	352.29 (279.15– 429.23)	757133.50 (579855.70– 943158.90)	431.32 (330.33– 537.30)	0.7 (0.65– 0.74)	<0.001	346.46 (326.20– 365.43)	0.19 (0.18– 0.20)	275.00 (252.28– 287.28)	0.16 (0.14– 0.16)	–0.66 (–0.93 to –0.40)	<0.001
High-middle SDI	425481.90 (317589.50– 555225.20)	140.61 (104.95– 183.48)	528681.40 (385207.30– 693636.40)	208.35 (151.81– 273.35)	1.36 (1.29– 1.42)	<0.001	790.91 (734.78– 849.47)	0.26 (0.24– 0.28)	438.52 (404.15– 477.80)	0.17 (0.16– 0.19)	–1.38 (–1.71 to –1.05)	<0.001
Middle SDI	531993.10 (381600.90– 712833.40)	97.72 (70.09– 130.94)	831384.80 (596895.90– 1109552.80)	151.29 (108.62– 201.91)	1.52 (1.48– 1.55)	<0.001	2082.58 (1711.62– 2319.35)	0.38 (0.31– 0.43)	1626.37 (1451.29– 1789.24)	0.30 (0.26– 0.33)	–0.88 (–1.00 –0.76)	<0.001
Low-middle SDI	517355.80 (364377.20– 696408.30)	146.19 (102.96– 196.79)	896109.80 (635298.40– 1198235.00)	175.56 (124.46– 234.75)	0.63 (0.61– 0.65)	<0.001	1663.30 (1175.90– 2016.18)	0.47 (0.33– 0.57)	2149.56 (1759.92– 2542.15)	0.42 (0.34– 0.50)	–0.40 (–0.71 to –0.08)	0.013
Low SDI	254698.50 (178945.30– 346061.80)	155.45 (109.21– 211.21)	629520.10 (443934.10– 853197.60)	169.46 (119.50– 229.67)	0.30 (0.29– 0.31)	<0.001	810.81 (584.60– 1011.82)	0.49 (0.36– 0.62)	1627.04 (1352.23– 1913.92)	0.44 (0.36– 0.52)	–0.41 (–0.55 to –0.28)	<0.001
African Region	246283.00 (173554.90– 332447.10)	151.65 (106.87– 204.70)	574927.90 (405340.60– 778134.00)	160.79 (113.36– 217.61)	0.20 (0.20– 0.21)	<0.001	756.66 (572.35– 933.22)	0.47 (0.35– 0.57)	1426.96 (1163.25– 1703.24)	0.40 (0.33– 0.48)	–0.53 (–0.56 to –0.49)	<0.001
Eastern Mediterranean Region	188344.40 (134495.50– 251883.70)	156.54 (111.79– 209.36)	528028.50 (380416.60– 702236.60)	249.93 (180.06– 332.38)	1.63 (1.61– 1.66)	<0.001	445.83 (369.09– 520.81)	0.37 (0.30– 0.43)	908.50 (715.86– 1128.36)	0.43 (0.34– 0.53)	0.55 (0.36– 0.74)	<0.001
European Region	415494.20 (318536.80– 524672.40)	211.76 (162.34– 267.40)	547017.10 (399042.30– 708770.90)	336.14 (245.21– 435.53)	1.62 (1.58– 1.65)	<0.001	516.76 (489.00– 549.06)	0.26 (0.25– 0.28)	363.33 (331.63– 396.74)	0.22 (0.20– 0.24)	–0.54 (–1.11 to 0.03)	0.06
Region of the Americas	500181.10 (381571.30– 631143.30)	253.04 (193.03– 319.29)	643031.90 (483735.10– 815315.50)	277.95 (209.10– 352.42)	0.32 (0.24– 0.40)	<0.001	630.59 (590.42– 738.95)	0.32 (0.30– 0.37)	742.88 (596.40– 822.36)	0.32 (0.26– 0.36)	0.04 (–0.21 to 0.30)	0.73
South-East Asia Region	578152.60 (408841.40– 780566.70)	144.38 (102.10– 194.93)	940947.80 (666752.10– 1276630.30)	168.25 (119.22– 228.27)	0.53 (0.52– 0.54)	<0.001	1936.56 (1279.28– 2370.03)	0.48 (0.32– 0.59)	2077.30 (1666.33– 2481.30)	0.37 (0.30– 0.44)	–0.96 (–1.42 to –0.49)	<0.001
Western Pacific Region	443476.40 (335771.80– 572501.30)	94.79 (71.76– 122.36)	401897.50 (298538.90– 517112.00)	119.94 (89.10– 154.33)	0.80 (0.76– 0.84)	<0.001	1402.94 (1076.95– 1552.13)	0.30 (0.23– 0.34)	592.61 (524.44– 658.22)	0.18 (0.16– 0.20)	–1.77 (–1.98 to –1.55)	<0.001

Data in parentheses are 95% uncertainty intervals for cases, prevalence, and mortality, and 95% CIs for AAPCs.

*AAPC* average annual percentage change.

### Global burdens of T1D in adolescents and young adults by sex

In the context of sex, among the 3,644,613 T1D cases among adolescents and young adults globally in 2019, 1,871,433 (53·91%) occurred in boys. Both sexes exhibited a significant increase in T1D prevalence and incidence over the 30-year period. T1D incidence in boys increased from 65,053·33 cases (95% UI, 44,514·79–87,999·81) in 1990 to 111,120·71 cases (95% UI, 75,271·16–153,830·34) in 2019, representing a 70·85% increase. T1 incidence in girls increased from 55,526·95 cases (95% UI, 37,333·90–75,781·23) in 1990 to 95,015·41 cases (95% UI, 63,469·04–132,623·84) in 2019, representing a 71·12% increase. In terms of T1D prevalence in boys, an increase from 1,206,677 cases (95% UI, 891,833·4–1,571,014) in 1990 to 1,871,433 cases (95% UI, 1,360,287·7–2,446,297) in 2019 was observed, representing a 55·09% increase. T1D prevalence in girls increased from 1,169,767 cases (95% UI, 865,724·6–1,507,234) in 1990 to 1,773,180 cases (95% UI, 1,288,764·3–2,325,674) in 2019, representing a 51·58% increase over the three decades. While the global DALY rate in boys increased (APCC, 0·28 [95% CI, 0·12–0·43]), both global mortality (APCC, -0·87 [95% CI, -1·00–-0·74]) and DALY (-0·43 [95% CI, -0·57–-0·28]) rates decreased among girls.

### Global burdens of T1D in adolescents and young adults by age group

T1D trends among adolescents and young adults varied according to age. Globally, the most rapidly increasing in T1D incidence (AAPC, 1·78 [95% CI, 1·65–1·91]) and prevalence (AAPC, 0·96 [95% CI, 0·91–1·01]) over the past 30 years were observed in young adults aged 20–24 years. Despite the increase in T1D incidence and prevalence among all three age subgroups, mortality in all subgroups decreased. The largest decline in the T1D mortality rate between 1990 and 2019 was observed among older adolescents aged 15–19 years (from 0·34 deaths per 100,000 population [95% UI, 0·26–0·40] to 0·29 deaths per 100,000 population [95% UI, 0·24–0·33]; AAPC, -0·64 [95% CI, -0·88–-0·40]). While an increase in the DALY rate was observed among young adults aged 20–24 years (from 44·25 per 100,000 population [95% UI, 36·56–51·53] to 45·76 per 100,000 population [95% UI, 38·96–53·85]; AAPC, 0·14 [95% CI, 0·04–0·24]), a decrease was observed among those aged 10–14 years (from 25·44 per 100,000 population [95% UI, 18·23–31·77] to 23·82 per 100,000 population [95% UI, 19·36–29·81]; AAPC, -0·23 [95% CI, -0·48–0·03]) and 15–19 years (from 32·51 per 100,000 population [95% UI, 24·95–38·91] to 30·70 per 100,000 population [95% UI, 25·08–37·55]; AAPC, -0·22 [95% CI, -0·39–-0·04]) between 1990 and 2019.

### Global burdens of T1D in adolescents and young adults by SDI

T1D burden among adolescents and young adults differed substantially according to the SDI. Countries with high SDI had the highest T1D prevalence (431·32 cases per 100,000 population [95% UI, 33·33–537·30]; AAPC, 0·70 [95% CI, 0·65–0·74]) and incidence (21·53 cases per 100,000 population [95% UI, 14·55–29·53]; AAPC, 1·25 [95% CI, 1·17–1·33]) rates in 2019, but the lowest mortality rate (0·16 deaths per 100,000 population [95% UI, 0·14–0·16]; AAPC, -0·66 [95% CI, -0·93–-0·40]). Notably, countries with a middle SDI had the lowest T1D prevalence (151·29 cases per 100,000 population [95% UI, 108·62–201·91]; AAPC, 1·52 [95% CI, 1·48–1·55]) and incidence (8·58 per 100,000 population [95% UI, 5·73–11·95]; AAPC, 1·79 [95% CI, 1·74–1·84]) rates in 2019. Countries with a low SDI had the highest mortality (0·44 deaths per 100,000 population [95% UI, 0·36–0·52]; AAPC, -0·41 [95% CI, -0·55–-0·28]) and DALY (39·73 per 100,000 population [95% UI, 32·90–47·47]; AAPC, -0·27 [95% CI, -0·38–-0·15]) rates in 2019. Over the past 30 years, both T1D prevalence and incidence increased across all SDI quintiles, whereas mortality rates decreased. All countries exhibited a reduction in the DALY rate from 1990 to 2019, with the exception of those countries categorized in the high SDI quintile group.

### Regional and national burdens of T1D in adolescents and young adults

When classified according to World Health Organization (WHO) regions, the European region exhibited the most rapid increase in T1D incidence rate of T1D for adolescents and young adults between 1990 and 2019 (from 11·18 cases per 100,000 population [95% UI, 8·24–14·52] to 18·80 cases per 100,000 population [95% UI, 12·54–25·90]; AAPC, 1·81 [95% CI, 1·76–1·86]). The highest mortality rate in 2019 was observed in the Eastern Mediterranean region (0·43 deaths per 100,000 population [95% UI, 0·34–0·53]), followed by the African region (0·40 deaths per 100,000 population [95% UI, 0·33–0·48]). The Western Pacific region showed the lowest incidence (5·47 cases per 100,000 population [95% UI, 3·59–7·63]), prevalence (119·94 cases per 100,000 population [95% UI, 89·10–154·33]), mortality (0·18 deaths per 100,000 population [95% UI, 0·16–0·20]), and DALY (24·61 per 100,000 population [95% UI, 20·83–28·77]) rates in 2019. The African region exhibited a modest increase in T1D incidence rate (from 10·05 cases per 100,000 population [95% UI, 6·73–13·93] to 10·60 cases per 100,000 population [95% UI, 7·07–14·87]; AAPC, 0·18 [95% CI, 0·17–0·20]) and prevalence (from 151·65 cases per 100,000 population [95% UI, 106·87–204·70] to 160·79 cases per 100,000 population [95% UI, 113·36–217·61]; AAPC, 0·20 [95% CI, 0·20–0·21]) rates between 1990 and 2019.

**Fig. 2:**
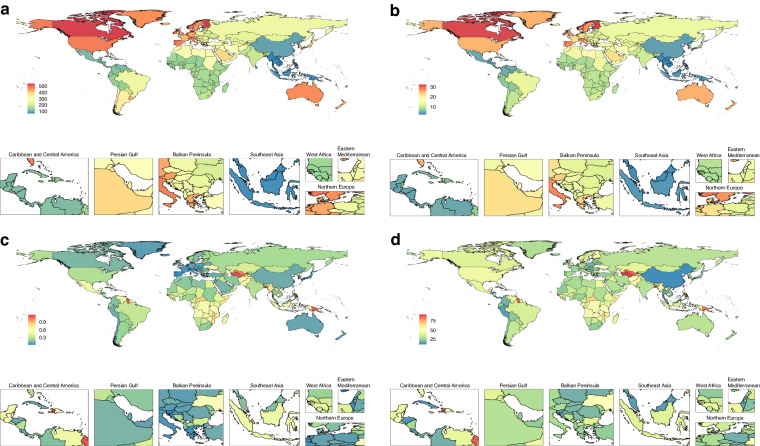
Global trends in T1D among adolescents in 204 countries and territories. Global map of 2019 prevalence (**a**), incidence (**b**), deaths (**c**), and DALYs (**d**) of T1D among adolescents from 1990 to 2019. DALY disability-adjusted life year.

At the national level, Finland had the highest T1D incidence rate among adolescents and young adults in 2019 (32·56 cases per 100,000 population [95% UI, 22·41–44·59]; AAPC, -0·37 [95% CI, -0·78–0·04]), followed by Canada (31·89 cases per 100,000 population [95% UI, 21·83–44·01]; AAPC, 0·36 [95% CI, 0·20–0·53]) (Fig. 2, Supplementary Tables 1–2). The Solomon Islands (1·20 deaths per 100,000 population [95% UI, 0·77–1·73]; AAPC, 0·75 [95% CI, 0·47–1·03]) had the highest mortality rate in 2019, followed by Turkmenistan (1·15 deaths per 100,000 population [95% UI, 0·90–1·45]; AAPC, 2·12 [95% CI, 1·21–3·03]) (Supplementary Table 3). Despite the declining trend in the global DALY rate due to T1D, rates remained high in countries with a low SDI; Turkmenistan had the highest DALY rate in 2019 (AAPC, 1·95 [95% CI, 1·17–2·73]), followed by Haiti (87·44 per 100,000 population [95% UI, 57·16–119·46]) (Supplementary Table 4).

## Discussion

This study provided an updated and comprehensive evaluation of global, regional, and national T1D burden among adolescents and young adults aged 10–24 years old, based on data from the GBD Study 2019. Diabetes is the third most common disease in children and adolescents aged <18 years.^14^ Our study documented 206,136 new cases among young adult and adolescents worldwide in 2019, which led to 6123 deaths. In addition to systemic complications, T1D also has long-term effects on health-related quality of life and psychosocial functioning.^15,16^ Previous studies have shown that the global T1D mortality rate among adolescents has decreased with improvements in diagnosis and treatment planning.^17^ While we observed an overall decrease in global T1D mortality rate among adolescents between 1990 and 2019 (AAPC, –0·41 [95% CI, –0·60–-0·22]), there was also an increase between 1990 and 1999 (AAPC, 0·69 [95% CI, 0·56–0·82]). Our findings provide further insight into the global burden of the T1D epidemic among adolescents and young adults and highlight the need for government action to improve diabetes management for this age group.

Notably, our use of the SDI demonstrated a close association between T1D burden and socioeconomic development.^18^ Inadequate T1D diagnosis and treatment are likely to be major contributors to early mortality, especially in low-SDI countries.^19^ Our study showed that adolescents in countries with a greater SDI exhibited higher T1D incidence and prevalence rates in 2019, whereas middle-SDI countries had the lowest incidence and prevalence rates. Prior studies have shown that low socioeconomic status is associated with higher mortality and morbidity in adults with T1D, even in those who are capable of accessing a universal healthcare system.^20^ Similarly, our results indicated that the T1D mortality rate among individuals aged 10–24 years was highest in low-SDI countries. We also found that countries with a low SDI had the highest DALY rate, despite a global declining trend among adolescents between 1990 and 2019.

Previous studies have attributed geographic differences in T1D prevalence and clinical characteristics to inherent variations among ethnic groups and migration between countries.^21^ Our results showed that European countries had the highest T1D incidence among adolescents in 2019, whereas countries in the Western Pacific region had the lowest incidence. Prior studies have also found that T1D incidence among children aged 0–14 years differs between Nordic countries and their neighboring countries and that this may be related to differences in population density.^22^ A study that examined T1D incidence and trends in the 15–39-year-old age group between 1992 and 1996 in Finland concluded that the risk of T1D extended into young adulthood.^23^

Nonetheless, we observed that adolescents with T1D in low-SDI or developing countries tended to have higher mortality and DALY rates, which may have been due to a lack of financial support and poor diabetes management. Our results showed that the Solomon Islands had the highest T1D mortality rate in 2019, followed by Turkmenistan and Guyana. Diabetes is currently the fourth-leading cause of death in Guyana, South America.^24^ Multiple risk factors have been implicated in the increasing incidence of T1D among adolescents.^25^ Environmental factors, including childhood obesity, chronic viral infections, and maternal-child interactions, have been considered to be responsible for the current evolving pattern of T1D incidence.^26^ Previous studies have reported that adversities during childhood may increase the risk of T1D through hyperactivation of the stress response system, especially in individuals exposed to increasing annual rates of childhood adversities.^27^

There is strong evidence that sex plays an important role in T1D incidence among adolescents and young adults. Previous studies have shown that T1D incidence among children is higher in males than in females.^28^ A Finnish study that analyzed 3,277 children (<10 years old) diagnosed with T1D reported that boys more often had insulin autoantibody-initiated autoimmunity, whereas glutamic acid decarboxylase-initiated autoimmunity was observed more frequently in girls.^29^ In our study, the AAPC among females showed a declining trend compared to males, thus indicating that more attention should be paid to diabetes care for young males.

We found that age was an important factor affecting differences in the burden of T1D among adolescents and young adults. A US study estimating the total number of youth aged under 20 years with diabetes reported that T1D prevalence increased with age.^30^ Analysis of a national registry that included 505 hospitals in China found that the peak incidence per 100,000 person-years occurred in the 10–14-year-old age group.^31^ Consistent with previous studies, we observed that younger adolescents aged 10–14 years had the highest T1D incidence rate in 2019; the prevalence rate in young adults aged 20–24 years was 246·79 cases per 100,000 population, nearly twice that of younger adolescents aged 10–14 years (143·19 cases per 100,000 population). Notably, the mortality rate of T1D in 2019 among young adults was 0·49 deaths per 100,000 population, more than double that of those aged 10–14 years (0·22 deaths per 100,000 population). This is pertinent, as managing T1D in adolescents is challenging from both medical and psychosocial perspectives, due to the vulnerable period during which parental caretaking is normative and adolescent behavior is unpredictable.^32^

Compared with a previous GBD study that analyzed diabetes burden,^33,34^ we provided a more comprehensive and specific analysis of T1D among adolescents and young adults aged 10–24 years and identified the most prominent changes in global trends by using Joinpoint regression analysis. Nevertheless, our study had several limitations. First, our results were based on the GBD Study 2019, which collected data from 1990 to 2019 and included countries with large boundaries. This may have been a source of significant variation in our results. Second, the determination of T1D burden in the GBD Study 2019 may have been affected by the detection method used, screening quality, and availability of local medical resources. Thus, T1D burden in countries with a low SDI tended to be underestimated, which could have introduced bias into our results. Third, while ethnic factors have been reported to affect the distribution of T1D among adolescents, these parameters were not evaluated in the GBD Study 2019.

## Conclusion

T1D among adolescents and young adults is a growing global health problem, especially in countries with a low SDI and less well-developed economy. The global burden of T1D among adolescents is substantial, although mortality and DALY rates have declined over the past 30 years due to advances in healthcare. As the incidence and severity of T1D among adolescents and young adults are still increasing, there is an urgent need for the implementation of lifestyle modification programs in childhood.

## Supplementary information

Supplementary Tables

## Data Availability

All data used in this study can be freely accessed at the GBD 2019 study (https://vizhub.healthdata.org/gbd-results).
