# Supplementary material for: Global, regional, and national burden of type 1 diabetes in adolescents and young adults
Source: Pediatr Res. 2024 Mar 5;97(2):568–76. doi: 10.1038/s41390-024-03107-5 (PMC12015108; doi:10.1038/s41390-024-03107-5)
Supplement: Supplementary file 1 — Supplementary Tables [file 41390_2024_3107_MOESM1_ESM.pdf]

Supplementary table 1. The top 10 countries with highest incidence of T1D among adolescents and young adults in the world in 1990 and their AAPCs from 1990 to 2019.

National	Incidence,1990 Rates, per 100,000	95%UI	Incidence,2019 Rates, per 100,000	95%UI	AAPC	P value
Finland	35.57	34.16-37.03	32.56	22.41-44.59	-0.37 (-0.78-0.04)	0.07
Canada	28.38	25.31-31.80	31.89	21.83-44.01	0.36 (0.20-0.53)	<0.001
Norway	24.54	16.73-33.69	29.48	19.34-41.41	0.67 (0.61-0.74)	<0.001
Spain	14.22	12.16-16.45	28.25	19.00-39.16	2.34 (2.20-2.48)	<0.001
Italy	21.17	14.18-29.51	27.89	18.55-38.97	0.95 (0.56-1.35)	<0.001
Ireland	13.33	8.66-19.46	27.65	18.87-37.56	1.91 (1.81-2.00)	<0.001
Malta	17.57	11.79-24.45	27.53	18.62-38.24	1.57 (1.51-1.64)	<0.001
Sweden	24.81	16.66-34.11	27.28	18.13-38.21	0.33 (0.25-0.41)	<0.001
Andorra	16.14	10.80-22.23	27.10	18.69-37.52	1.81 (1.66-1.96)	<0.001
Switzerland	16.10	10.80-22.29	26.51	17.96-36.61	1.73 (1.69-1.78)	<0.001

Abbreviations: AAPC, average annual percentage change; Data in parentheses are 95% uncertainty intervals for cases, incidences, and DALYs, and 95% CIs for AAPCs.

Supplementary table 2. The top 10 countries with highest prevalence of T1D among adolescents and young adults in the world in 1990 and their AAPCs from 1990 to 2019.

National	Prevalence,1990 Rates, per 100,000	95%UI	Prevalence,2019 Rates, per 100,000	95%UI	AAPC	P value
Finland	673.97	647.89-701.92	542.66	402.91-680.97	-0.80 (-1.05 to -0.55)	<0.001
Canada	562.78	507.12-624.67	539.13	398.63-686.84	-0.15 (-0.35 to 0.05)	0.13
Norway	425.65	309.75-541.77	489.52	357.63-630.85	0.54 (0.49 to 0.6)	<0.001
Malta	296.86	215.19-398.43	488.34	357.62-618.64	1.73 (1.65 to 1.81)	<0.001
United States of America	487.20	365.40-612.82	479.16	361.98-597.90	-0.05 (-0.12 to 0.01)	0.10
Spain	277.81	242.97-315.64	478.93	346.90-611.60	1.88	<0.001

					(1.83 to 1.93)	
Italy	409.71	297.35-529.59	474.96	347.58-609.80	0.54 (0.16 to 0.91)	0.01
Switzerland	300.77	218.70-405.09	473.56	347.01-604.18	1.59 (1.55 to 1.63)	<0.001
Andorra	295.86	212.80-397.11	473.55	344.09-601.35	1.64 (1.55 to 1.72)	<0.001
Ireland	255.08	205.65-315.38	469.39	342.37-593.06	2.15 (2.03 to 2.27)	<0.001

Abbreviations: AAPC, average annual percentage change; Data in parentheses are 95% uncertainty intervals for cases, incidences, and DALYs, and 95% CIs for AAPCs.

Supplementary table 3. The top 10 countries with highest mortality of T1D among adolescents and young adults in the world in 1990 and their AAPCs from 1990 to 2019.

**National**	**Deaths,1990 Rates, per 100,000**	**95%UI**	**Deaths,2019 Rates, per 100,000**	**95%UI**	**AAPC**	**P value**
Solomon Islands	0.97	0.64-1.45	1.20	0.77-1.73	0.75 (0.47 to 1.03)	<0.001
Turkmenistan	0.63	0.55-0.74	1.15	0.90-1.45	2.12 (1.21 to 3.03)	<0.001
Guyana	1.17	0.88-1.48	1.13	0.80-1.58	0.03 (-1.1 to 1.17)	0.96
Haiti	1.57	0.90-2.51	1.13	0.67-1.57	-0.97 (-1.21 to -0.72)	<0.001
Papua New Guinea	1.00	0.64-1.53	1.13	0.75-1.66	0.34 (0.24 to 0.44)	<0.001
Uzbekistan	0.47	0.40-0.53	1.05	0.82-1.28	2.86 (2.23 to 3.49)	<0.001
Fiji	1.11	0.78-1.52	1.00	0.71-1.38	-0.33 (-0.61 to -0.04)	0.03
Kiribati	0.95	0.67-1.31	0.96	0.63-1.44	0.00 (-0.1 to 0.1)	1.00
Bangladesh	1.07	0.58-1.51	0.92	0.59-1.31	-0.43 (-0.63 to -0.22)	<0.001
Saint Vincent and the Grenadines	0.97	0.76-1.18	0.84	0.65-1.05	-0.47 (-1.41 to 0.48)	0.33

Abbreviations: AAPC, average annual percentage change; Data in parentheses are 95% uncertainty intervals for cases, incidences, and DALYs, and 95% CIs for AAPCs.

Supplementary table 4. The top 10 countries with highest DALYs of T1D among adolescents and young adults in the world in 1990 and their AAPCs from 1990 to 2019.

National	DALYs,1990 Rates, per 100,000	95%UI	DALYs,2019 Rates, per 100,000	95%UI	AAPC	P value
Turkmenistan	52.73	45.94-61.06	91.88	73.09-113.42	1.95 (1.17 to 2.73)	<0.001
Haiti	116.42	70.57-181.94	87.44	57.16-119.46	-0.83 (-1.06 to -0.6)	<0.001
Solomon Islands	71.25	47.92-103.76	86.61	57.22-123.51	0.71 (0.45 to 0.97)	<0.001
Guyana	87.89	67.42-109.72	86.30	64.29-115.51	-0.04 (-1.23 to 1.18)	0.95
Uzbekistan	41.25	35.11-47.52	84.67	67.33-102.88	2.57 (2.01 to 3.14)	<0.001
Papua New Guinea	73.40	47.64-109.52	82.08	56.04-118.47	0.34 (0.24 to 0.43)	<0.001
Bangladesh	84.59	49.11-115.68	76.08	50.34-106.58	-0.28 (-0.46 to -0.1)	0.003
Fiji	82.42	58.75-113.52	74.57	54.29-101.67	-0.31 (-0.58 to -0.04)	0.03
Kiribati	70.48	50.78-95.32	71.23	48.70-104.08	0.04 (-0.08 to 0.15)	0.56
Central African Republic	71.11	51.60-96.37	66.72	47.60-95.96	-0.26 (-0.71 to 0.19)	0.25

Abbreviations: AAPC, average annual percentage change; Data in parentheses are 95% uncertainty intervals for cases, incidences, and DALYs, and 95% CIs for AAPCs.
